# Exploiting the Diversity of Saccharomycotina Yeasts To Engineer Biotin-Independent Growth of Saccharomyces cerevisiae

**DOI:** 10.1128/AEM.00270-20

**Published:** 2020-06-02

**Authors:** Anna K. Wronska, Meinske P. Haak, Ellen Geraats, Eva Bruins Slot, Marcel van den Broek, Jack T. Pronk, Jean-Marc Daran

**Affiliations:** a Department of Biotechnology, Delft University of Technology, Delft, The Netherlands; University of Buenos Aires

**Keywords:** fungal biotin synthesis, vitamin B_7_, *BIO1*, *de novo* synthesis, oxygen-requiring enzyme, biotin, *Cyberlindnera fabianii*, *Saccharomyces cerevisiae*, Saccharomycotina, metabolic engineering, oxygen requirement, prototrophy

## Abstract

Biotin, an important cofactor for carboxylases, is essential for all kingdoms of life. Since native biotin synthesis does not always suffice for fast growth and product formation, microbial cultivation in research and industry often requires supplementation of biotin. *De novo* biotin biosynthesis in yeasts is not fully understood, which hinders attempts to optimize the pathway in these industrially relevant microorganisms. Previous work based on laboratory evolution of Saccharomyces cerevisiae for biotin prototrophy identified Bio1, whose catalytic function remains unresolved, as a bottleneck in biotin synthesis. This study aimed at eliminating this bottleneck in the S. cerevisiae laboratory strain CEN.PK113-7D. A screening of 35 Saccharomycotina yeasts identified six species that grew fast without biotin supplementation. Overexpression of the S. cerevisiae *BIO1* (*ScBIO1*) ortholog isolated from one of these biotin prototrophs, Cyberlindnera fabianii, enabled fast growth of strain CEN.PK113-7D in biotin-free medium. Similar results were obtained by single overexpression of *C. fabianii BIO1* (*CfBIO1*) in other laboratory and industrial S. cerevisiae strains. However, biotin prototrophy was restricted to aerobic conditions, probably reflecting the involvement of oxygen in the reaction catalyzed by the putative oxidoreductase *Cf*Bio1. In aerobic cultures on biotin-free medium, S. cerevisiae strains expressing *Cf*Bio1 showed a decreased susceptibility to contamination by biotin-auxotrophic S. cerevisiae. This study illustrates how the vast Saccharomycotina genomic resources may be used to improve physiological characteristics of industrially relevant S. cerevisiae.

**IMPORTANCE** The reported metabolic engineering strategy to enable optimal growth in the absence of biotin is of direct relevance for large-scale industrial applications of S. cerevisiae. Important benefits of biotin prototrophy include cost reduction during the preparation of chemically defined industrial growth media as well as a lower susceptibility of biotin-prototrophic strains to contamination by auxotrophic microorganisms. The observed oxygen dependency of biotin synthesis by the engineered strains is relevant for further studies on the elucidation of fungal biotin biosynthesis pathways.

## Abstract (precis)

The reported metabolic engineering strategy to enable optimal growth in the absence of biotin is of direct relevance for large-scale industrial applications of S. cerevisiae. Important benefits of biotin prototrophy include cost reduction during the preparation of chemically defined industrial growth media as well as a lower susceptibility of biotin-prototrophic strains to contamination by auxotrophic microorganisms. The observed oxygen dependency of biotin synthesis by the engineered strains is relevant for further studies on the elucidation of fungal biotin biosynthesis pathways.

## INTRODUCTION

Biotin is a class B vitamin (B_7_) and an essential cofactor for higher eukaryotes, such as mammals and birds, which have to acquire it from their diet (1). In contrast, most prokaryotes, plants, and some fungi can synthesize biotin (2). In Saccharomyces cerevisiae, biotin serves as an important cofactor for enzymes catalyzing carbon dioxide transfers: the cytosolic (AccI) and mitochondrial (Hfa1) acetyl-coenzyme A (CoA) carboxylases, generating malonyl-CoA for fatty acid synthesis (3), as well as the pyruvate carboxylases (Pyc1 and Pyc2), responsible for anaplerotic formation of oxaloacetate (4). In addition, biotin is involved in the reaction catalyzed by urea amidolyase (Dur1 and Dur2), which releases ammonia and carbon dioxide from urea (5). The only biotin-dependent protein in S. cerevisiae that is not a carboxylase is Arc1, which is involved in tRNA aminoacylation (6).

A fast, widely applicable metabolic engineering strategy for the construction of fully biotin-prototrophic yeast strains has significant economic and technological relevance. The omission of biotin from medium formulations would enable the design of less expensive, easier-to-handle media with a longer shelf life. Such advantages have been reported for heterologous protein production with a Komagatella phaffi (formerly known as Pichia pastoris) strain expressing the S. cerevisiae biotin biosynthesis genes *BIO1* and *BIO6*. However, this biotin-prototrophic strain exhibited slower growth in the absence of biotin than in its presence (7). Additionally, the use of fully biotin-prototrophic strains of yeast in fermentation processes that do not contain biotin may reduce the impact of contamination with wild yeast or even bacterial strains that grow slower or not at all in the absence of this cofactor (8–10).

The genomes of most S. cerevisiae strains carry all genes necessary to encode all known enzymes in the yeast biotin synthesis pathway. In some cases, S. cerevisiae strains lack one or more biosynthetic genes, resulting in full biotin auxotrophy (e.g., the reference strain S288C is missing both *BIO1* and *BIO6*). In the intensively studied S. cerevisiae strain CEN.PK113-7D (11–13), all biotin pathway genes are present (14). Nevertheless, the cultivation of this strain on synthetic medium devoid of biotin results in a much lower specific growth rate than cultivation on biotin-containing medium (14, 15). Growth defects in the absence of biotin were observed for several S. cerevisiae strains as well as for other budding yeasts. The extent to which growth was impaired was hypothesized to be dependent on the *BIO1* and *BIO6* gene cluster copy number of the specific strain (16). Evolutionary engineering of strain CEN.PK113-7D for biotin-independent growth enabled the selection of mutants whose growth in biotin-free medium was as fast as the growth of the reference strain in the presence of biotin. This evolved phenotype was linked to a 20- to 40-fold amplification of the *BIO1* and *BIO6* gene cluster originally located on chromosome I. Karyotyping and genome sequencing revealed that extensive chromosomal rearrangements and the formation of neochromosomes contributed to this amplification (15). Although these experiments showed that fast biotin-independent growth of S. cerevisiae is possible, the responsible mutations could not be easily transferred to other strains. Attempts to overexpress either the *BIO1* and *BIO6* cluster or *BIO1* alone only yielded a partial growth improvement in biotin-free media. This partial success in reverse engineering of biotin prototrophy indicates that additional mutations in genes responsible for intracellular transport or production of biotin synthesis intermediates are necessary. Furthermore, codon usage of *BIO* genes might be suboptimal, since these genes have been proposed to be acquired by horizontal gene transfer from bacteria (16). To date, the only option to obtain full biotin prototrophy in S. cerevisiae is to perform laboratory evolution experiments, which are time-consuming and lead to massive chromosomal rearrangements that might result in mutations that could negatively affect other cellular functions (17).

**FIG 1:**
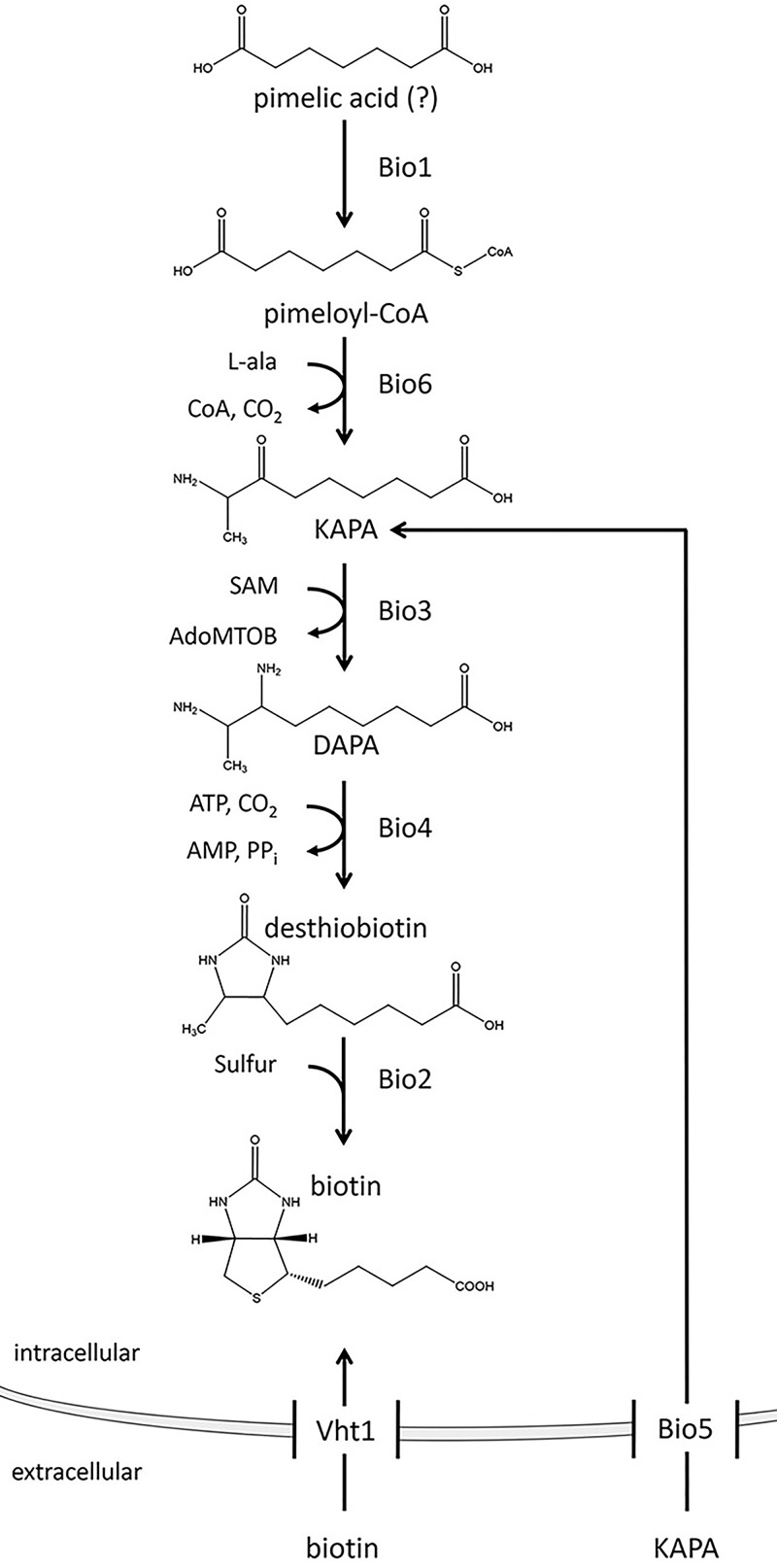
Yeast biotin biosynthesis pathway. The origin of pimelic acid, the substrate of the first reaction catalyzed by Bio1, is unknown and yields pimeloyl-CoA, which is further metabolized to 7-keto-8-aminopelargonic acid (KAPA), 7,8-diaminopelargonic acid (DAPA), desthiobiotin, and biotin by the action of the enzymes Bio6, Bio3, Bio4, and Bio2, respectively. Biotin can be imported into the cell via Vht1. The intermediate KAPA can be imported by Bio5.

The genetic information required for the biosynthesis of the fused-ring structure of biotin (catalyzed by Bio3, Bio4, and Bio2) is well-characterized in S. cerevisiae (Fig. 1) (18, 19). In contrast, the origin of the pimeloyl moiety contributing to the valeric acid chain remains elusive (Fig. 1) (20). S. cerevisiae *BIO1* (*ScBIO1*) previously was hypothesized to act as a CoA ligase that activates free pimelic acid for biotin synthesis, similar to the enzyme encoded by *bioW* in Bacillus subtilis (21). The hypothetical product of such a ligase, pimeloyl-CoA, could be converted into 8-amino-7-oxononanoate by yeast KAPA synthase (Bio6) using l-alanine and pyridoxal-5-phosphate. However, no evidence is available for the utilization of free pimelic acid by yeast. The lack of knowledge on the origin of the pimeloyl moiety in S. cerevisiae so far has hindered further targeted molecular improvement of biotin synthesis.

The aim of this study was to explore new metabolic engineering solutions to enable fast, unimpaired growth of S. cerevisiae without biotin supplementation. To this end, a set of Saccharomycotina yeasts was screened for specific growth rate in biotin-free synthetic medium lacking biotin. After the identification of the best-performing species, the genome sequences of these yeasts were interrogated for *ScBIO1* orthologs. The identified orthologous genes were functionally characterized in S. cerevisiae CEN.PK113-7D and evaluated for their ability to support fast growth in the absence of biotin under aerobic as well as anaerobic conditions. A promising candidate gene was expressed in several industrial and laboratory S. cerevisiae strains, and its impact was evaluated. Furthermore, we investigated how the cultivation of an engineered biotin-prototrophic S. cerevisiae strain on biotin-free medium affected the impact of an intentional contamination with an auxotrophic strain.

## RESULTS

### Characterization of biotin requirement in Saccharomycotina yeasts.

**FIG 2:**
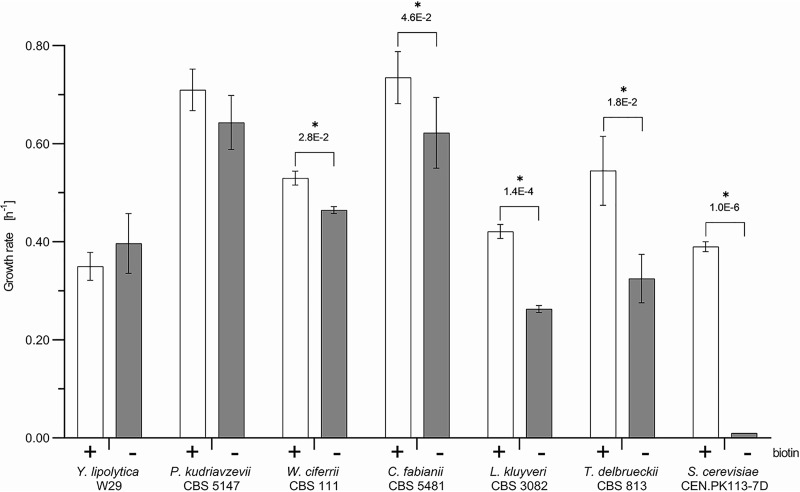
Specific growth rates of Saccharomycotina species in SMG (+; white bars) and biotin-free SMG (−; gray bars). The bars represent averages and standard deviations from three biological replicates. The data are derived from culture optical density (OD_660_) measurements over time during the exponential growth phase of shake flask batch cultures of Y. lipolytica W29, *P. kudriavzevii* CBS 5147, *W. ciferrii* CBS 111, *C. fabianii* CBS 5481, *L. kluyveri* CBS 3082, *T. delbrueckii* CBS 813, and S. cerevisiae CEN.PK113-7D. Statistical significance between growth rates in SMG and biotin-free SMG of each strain was determined using the Holm-Sidak method. An asterisk denotes comparison with *P* values lower than 5.0E−2.

The determination of nutritional requirements is a standard procedure in classical yeast taxonomy. While biotin requirements of individual species are reported in the yeast taxonomy reference book *The Yeasts: a Taxonomic Study* (22), this information is only qualitative. To capture quantitative information on biotin requirements, we determined the specific growth rates of a set of 35 biotechnologically relevant yeasts belonging to the Saccharomycotina subphylum in biotin-free synthetic medium (biotin-free SMG). Of these 35 species, eleven exhibited growth, albeit at different rates. We focused further analysis on fast-growing species, based on an arbitrary threshold specific growth rate of 0.25 h^−1^. Only strains belonging to the species Yarrowia lipolytica, Pichia kudriavzevii (syn. Candida krusei and *Issatchenkia orientalis*) (23), Wickerhamomyces ciferrii, Cyberlindnera fabianii (syn. *Candida fabianii*), Lachancea kluyveri, and Torulaspora delbrueckii met this criterion, with specific growth rates in biotin-free SMG ranging from 0.27 to 0.64 h^−1^. Specific growth rates of Y. lipolytica, *P. kudriavzevii*, and *C. fabianii* were not significantly higher in biotin-supplemented SMG. The other strains all showed lower specific growth rates in biotin-free medium than in biotin-supplemented media, but the growth rate did not fall below 60% of the growth rate on SMG supplemented with biotin (Fig. 2). Consistent with earlier observations (14–16), S. cerevisiae strain CEN.PK113-7D exhibited a specific growth rate of 0.39 ± 0.01 h^−1^ on biotin-supplemented SMG but grew extremely slowly (μ < 0.01 h^−1^) on biotin-free SMG.

### Identification of putative *ScBIO1* orthologs in Y. lipolytica, *P. kudriavzevii*, *W. ciferrii*, *C. fabianii*, *L. kluyveri*, and *T. delbrueckii*.

The growth performance of Y. lipolytica, *P. kudriavzevii*, *W. ciferrii*, *C. fabianii*, *L. kluyveri*, and *T. delbrueckii* in biotin-free SMG indicated that the Bio1 bottleneck in the biotin biosynthesis pathway of S. cerevisiae CEN.PK113-7D was absent in these species. Therefore, we hypothesized that biotin prototrophy of these strains is enabled by *ScBIO1* orthologs encoding enzymes with superior catalytic or regulatory properties.

**FIG 3:**
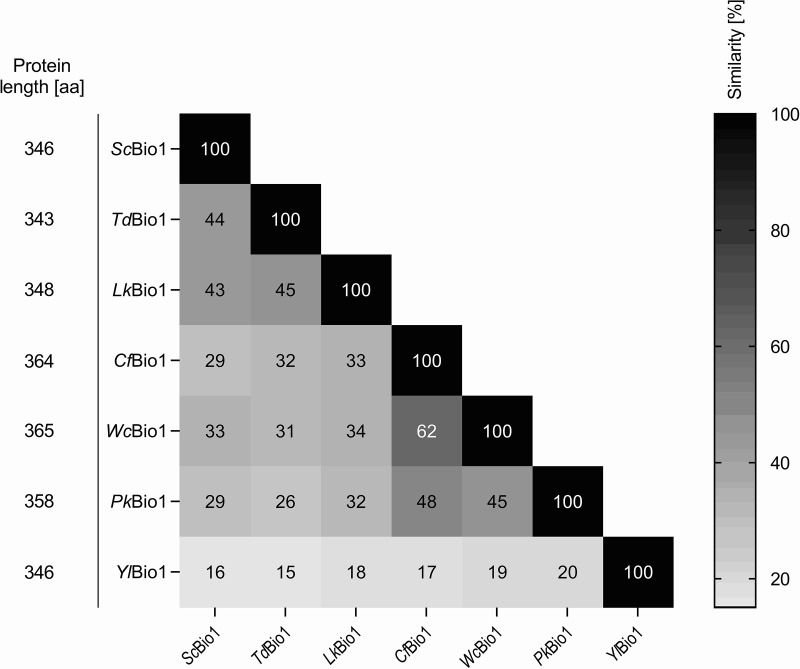
Amino acid sequence similarities of putative *Sc*Bio1 orthologs. Pair-wise alignments of amino acid sequences were generated using ClustalΩ (similarity matrix BLOSUM62). The percent amino acid sequence similarity between the orthologs is displayed in the intersecting cells. High amino acid similarity is indicated with black and low similarity with white. Protein length for each ortholog is represented as amino acids (aa) on the left.

Identification of putative *ScBIO1* orthologs was performed using the Basic Local Alignment Search Tool (BLAST) (https://blast.ncbi.nlm.nih.gov/Blast.cgi) (24), more specifically tBLASTn, which uses a protein sequence as the query against translation of a nucleotide database. Use of *Sc*Bio1 as a query revealed candidate proteins in five out of the six species (*P. kudriavzevii*, *W. ciferrii*, *L. kluyveri*, *T. delbrueckii*, and *C. fabianii*) but not in Yarrowia lipolytica (Table 1). Subsequently, the search was repeated with the *ScBIO1* orthologs found in *P. kudriavzevii*, *W. ciferrii*, *L. kluyveri*, *T. delbrueckii*, and *C. fabianii* as queries and using the Y. lipolytica W29 genome assembly as a database (BioProject accession no. PRJNA601425). Each of the five searches identified the same 346-amino-acid peptide sequence encoded by open reading frame YALI0A10010p (Table 1). Pair-wise comparisons of the identified putative Bio1 amino acid sequences using ClustalΩ (25) showed that the sequences of *L. kluyveri* Bio1 (*Lk*Bio1) and *T. delbrueckii* Bio1 (*Td*Bio1) showed the highest amino acid sequence similarities to the *Sc*Bio1 sequence (43 to 44%), while Y. lipolytica Bio1 (*Yl*Bio1) had the lowest similarity to the other homologs. Although its size matched perfectly with *Sc*Bio1, *Yl*Bio1 only showed 15% to 20% amino acid sequence similarity with the *Sc*Bio1 orthologous peptides (Fig. 3). Out of this set of putative Bio1 homologs, *C. fabianii* Bio1 (*Cf*Bio1) and *W. ciferrii* Bio1 (*Wc*Bio1) exhibited the highest pairwise amino acid similarity (62%), which is in line with the phylogenetic proximity of these yeast species (22).

**TABLE 1:** Identification of heterologous *BIO1* gene by tBLASTn and best hits for querying *Sc*Bio1, *Td*Bio1, *Lk*Bio1, *Cf*Bio1, *Wc*Bio1, or *Pk*Bio1 protein sequence

Species	Taxon identifier (NCBI)	Accession no.	Sequence	Open reading frame coordinates (bp)
*T. delbrueckii*	txid1076872	HE616748.1^*a*^	Chromosome 7	18080–19111
*L. kluyveri*	txid4934	AACE03000004.1^*a*^	Chromosome D SKLU-Cont10108	1095811–1094765
*C. fabianii*	txid36022	BCGI01000001.1^*a*^	Scaffold 0	1136478–1137572
*W. ciferrii*	txid1041607	CAIF01000264.1^*a*^	Contig 00264	39352–40449
*P. kudriavzevii*	txid4909	CP028531.1^*a*^	Chromosome 1	1085523–1084447
Y. lipolytica	txid284591	NC_006067.1^*b*^	Chromosome A	1097298–1096261

a Best hits for querying *Sc*Bio1 sequence.

b Best hits for querying *Td*Bio1, *Lk*Bio1, *Cf*Bio1, *Wc*Bio1, and *Pk*Bio1.

### Expression of the *BIO1* ortholog of *C. fabianii* enables fast growth of S. cerevisiae in biotin-free medium.

To investigate whether expression of the *ScBIO1* orthologs was able to promote the growth of S. cerevisiae CEN.PK113-7D in biotin-free medium, each *BIO1* gene was isolated from its respective genome and cloned under the transcriptional control of the S. cerevisiae *PYK1* promoter (*ScPYK1*p) and the S. cerevisiae *BIO1* terminator (*ScBIO1*t). Each expression module was integrated at the chromosomal *SGA1* locus of the *Spycas9*-expressing, CEN.PK-derived S. cerevisiae strain IMX585 by CRISPR/Cas9-mediated genome editing (26). The resulting strains, IMX1862 (*YlBIO1*↑), IMX1861 (*P. kudriavzevii BIO1*↑ [*PkBIO1*↑]), IMX1863 (*WcBIO1*↑), IMX1859 (*CfBIO1*↑), IMX1858 (*LkBIO1*↑), and IMX1857 (*TdBIO1*↑), and the control strain IMX1511 (*ScBIO1*↑), expressing the S. cerevisiae *BIO1* gene under the same regulatory sequences as the heterologous *BIO1* genes, were grown on biotin-containing and biotin-free SMG.

**FIG 4:**
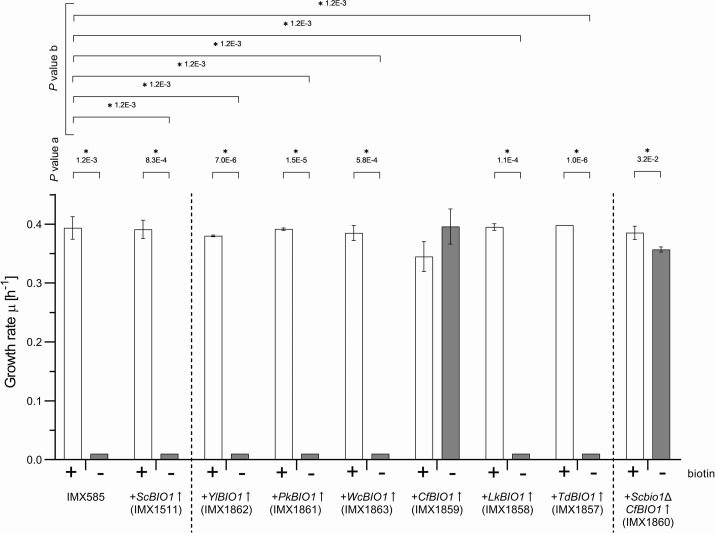
Specific growth rates of S. cerevisiae CEN.PK113-7D-derived strains expressing *ScBIO1* orthologs in SMG (+; white bars) and biotin-free SMG (−; gray bars). The bars represent averages and standard deviations from three biological replicates and are derived from measurement of culture optical density (OD_660_) over time during the exponential growth phase of shake flask batch cultures of strains IMX585, IMX1511 (*ScBIO1*↑), IMX1862 (*YlBIO1*↑), IMX1861 (*PkBIO1*↑), IMX1863 (*WcBIO1*↑), IMX1859 (*CfBIO1*↑), IMX1858 (*LkBIO1*↑), IMX1857 (*TdBIO1*↑), and IMX1860 (*Scbio1*Δ *CfBIO1*↑). Statistical significance between growth rates in SMG and biotin-free SMG of each strain was determined using the Holm-Sidak method, providing *P* value a. Statistical significance between the growth rates of IMX585 grown on SMG and the respective specific growth rates of engineered strains in SMG and biotin-free SMG was determined using the Holm-Sidak method, providing *P* value b. An asterisk denotes comparison with *P* values lower than 5.0E−2.

All strains expressing a heterologous *BIO1* ortholog grew on biotin-containing SMG at specific growth rates, ranging from 0.35 to 0.40 h^−1^, which were similar to the specific growth rate of the S. cerevisiae reference strain IMX585, which grew at 0.39 ± 0.01 h^−1^. As anticipated, strain IMX585 showed virtually no growth on biotin-free SMG after a third transfer to biotin-free SMG. The same was observed for strain IMX1511, which contained an expression cassette for S. cerevisiae *BIO1* (IMX1511) and for the S. cerevisiae strains expressing the *ScBIO1* orthologs of Y. lipolytica (IMX1862), *P. kudriavzevii* (IMX1861), *W. ciferrii* (IMX1863), *L. kluyveri* (IMX1858), and *T. delbrueckii* (IMX1857) (Fig. 4). Only strain IMX1859, which expressed the *C. fabianii BIO1* ortholog, showed growth in the third biotin-free SMG culture. Its specific growth rate of 0.40 ± 0.03 h^−1^ in the absence of biotin was not significantly different from the specific growth rate of the reference strain IMX585 on biotin-containing SMG (Fig. 4).

All strains expressing *ScBIO1* orthologs, including IMX1859, still harbored the native *ScBIO1* gene at its original locus. To verify that the expression of the *CfBIO1* gene sufficed to achieve full biotin prototrophy, the native *ScBIO1* copy was deleted in strain IMX1859, yielding strain IMX1860 (*Scbio1*Δ *CfBIO1*↑). The specific growth rate of IMX1860 in SMG was determined as 0.39 ± 0.01 h^−1^ and in biotin-free SMG as 0.36 ± 0.00 h^−1^, which is highly similar to the specific growth rates of strain IMX1859 on these media (Fig. 4). Despite a slight difference in biotin-containing and biotin-free media, the specific growth rates of strain IMX1860 indicated that *CfBIO1* was sufficient to confer S. cerevisiae strains from the CEN.PK lineage with the ability to grow fast in the absence of biotin.

### *C. fabianii BIO1* expression supports biotin prototrophy in multiple S. cerevisiae lineages.

**FIG 5:**
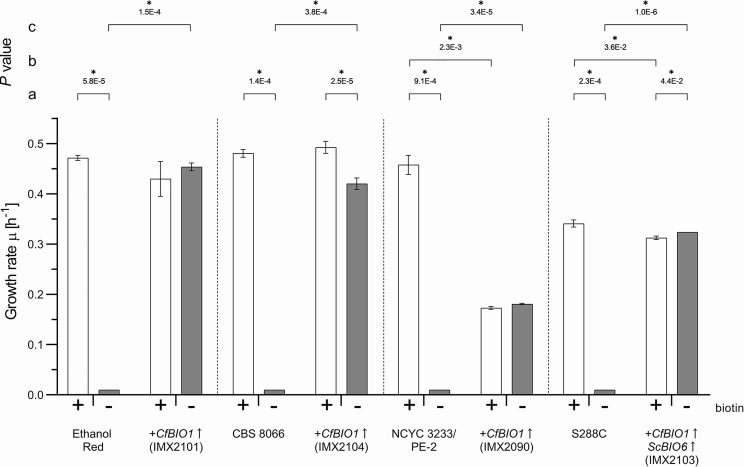
Specific growth rates of S. cerevisiae strains engineered for growth without biotin in SMG (+; black bars) and biotin-free SMG (−; white bars). The bars represent averages and standard deviations from three biological replicates, and specific growth rates were calculated from measurement of culture optical density (OD_660_) over time during the exponential growth phase of shake flask batch cultures of Ethanol Red, CBS 8066, NCYC 3233/PE-2, and S288C, as well as the respective engineered strains IMX2101 (Ethanol Red *CfBIO1*↑), IMX2104 (CBS 8066 *CfBIO1*↑), IMX2090 (NCYC 3233/PE-2 *CfBIO1*↑), and IMX2103 (S288C *CfBIO1*↑ *ScBIO6*↑). Statistical significance between growth rates in SMG and biotin-free SMG of each strain was determined using the Holm-Sidak method, providing *P* value a. Statistical significance between growth rates of the initial and the engineered strain on SMG was determined using the Holm-Sidak method, providing *P* value b. Statistical significance between growth rates of the initial and the engineered strain on biotin-free SMG was determined using the Holm-Sidak method, providing *P* value c. An asterisk denotes comparison with *P* values lower than 5.0E−2.

To investigate whether the results obtained in the laboratory CEN.PK strain lineage could be extrapolated to other genetic backgrounds, the *CfBIO1* expression cassette was expressed in the S. cerevisiae laboratory strains S288C and CBS 8066, in the Brazilian bioethanol strain NCYC 3233 (syn. PE-2), and in Ethanol Red (Lesaffre, France), a commercial S. cerevisiae strain used for first-generation bioethanol production. Prior to the expression of *CfBIO1*, none of these strains showed specific growth rates above 0.01 h^−1^ growth on biotin-free SMG. In biotin-containing SMG, strains NCYC 3233/PE-2, Ethanol Red, and CBS 8066 exhibited specific growth rates, ranging from 0.46 to 0.48 h^−1^, while S288C grew at 0.34 ± 0.01 h^−1^ (Fig. 5).

Prior to the integration of the *CfBIO1* expression cassette, genome sequences of these strains were checked for the presence of biotin biosynthetic genes *BIO2*, *BIO3*, and *BIO4*, as well as *BIO1* and *BIO6* (14). Except for S288C, all strains showed the presence of these five biosynthetic genes, suggesting that only *CfBIO1* suffices to restore biotin prototrophy. The reference S. cerevisiae S288C strain lacked the *ScBIO1* and *ScBIO6* genes, which usually occur as a two-gene cluster at the subtelomeric region of chromosome I (27). To complement the absence of *BIO6*, *CfBIO1* was cointegrated together with an expression cassette for *ScBIO6* in S288C.

Since, in contrast to the CEN.PK-derived strain IMX585, S. cerevisiae strains NCYC 3233/PE-2, Ethanol Red, CBS 8066, and S288C do not express Cas9, the integration of the *CfBIO1* cassette at the *SGA1* locus of these strains was achieved with plasmid pUDP145, which carries both the *ScSGA1* targeting genomic RNA (gRNA) and a *Spycas9* expression cassette. The *ScSGA1* Cas9-programmed double-strand break was repaired either with the *CfBIO1* expression module or, in the case of S288C, with two DNA fragments harboring *CfBIO1* and *ScBIO6* cassettes.

*Cf*Bio1-expressing Ethanol Red and CBS 8066 derivatives grew on biotin-containing SMG with the same specific growth rate as their parental strains. On biotin-free SMG, *CfBIO1* expression in Ethanol Red and CBS 8066 led to growth rates of 0.45 ± 0.01 h^−1^ and 0.42 ± 0.01 h^−1^, respectively, which corresponded to 105% and 85% of their specific growth rates in biotin-containing SMG (Fig. 5). In the NCYC 3233/PE-2 strain background, *CfBIO1* expression caused an unexpected decrease of its specific growth rate on SMG with biotin, from 0.46 ± 0.02 h^−1^ to 0.17 ± 0.00 h^−1^ (Fig. 5). The specific growth rate of strain S288C on SMG with biotin decreased slightly upon the combined expression of *CfBIO1* and *ScBIO6*. The cultivation of the NCYC 3233/PE-2- and S288-derived *CfBIO1*-expressing strains on biotin-free SMG yielded the same specific growth rates as those on biotin-containing SMG (Fig. 5).

### A biotin-prototrophic S. cerevisiae strain expressing *CfBIO1* outcompetes an auxotrophic strain.

**FIG 6:**
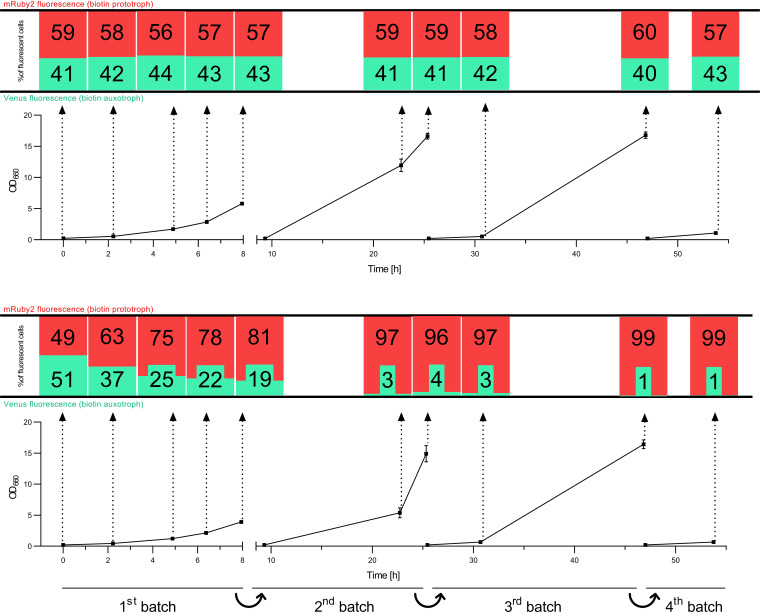
Culture optical density (OD_660_) and fluorescent population distribution of mRuby2-expressing strain IMX2238 (IMX585 *Scbio1*Δ *CfBIO1*↑; biotin prototroph) and Venus-expressing strain IMX2240 (IMX585 *Scbio1*Δ; biotin auxotroph) over time during cocultivation in four consecutive batch culture cycles on SMG (top) and biotin-free SMG (bottom). Both conditions were analyzed in two independent replicates each. Each OD_660_ time point was sampled and analyzed by flow cytometry. The distribution of mRuby2 (red bar) and Venus (green bar) fluorescent cells from the total of fluorescent cells (%) at each time point is represented in the bar graph above (connected by arrows).

**FIG 7:**
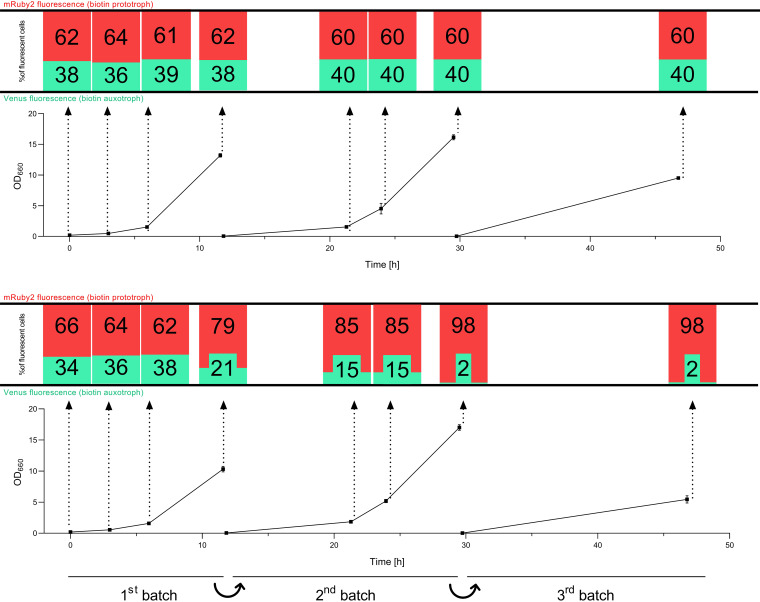
Culture optical density (OD_660_) and fluorescent population distribution of mRuby2-expressing strain IMX2238 (IMX585 *Scbio1*Δ *CfBIO1*↑; biotin prototroph) and Venus-expressing strain IMX2212 (IMX585; biotin auxotroph) over time during cocultivation in three consecutive batch culture cycles on SMG (top) and biotin-free SMG (bottom). Both conditions were analyzed in two independent replicates each. Each OD_660_ time point was sampled and analyzed by flow cytometry. The distribution of mRuby2 (red bar) and Venus (green bar) fluorescent cells from the total of fluorescent cells (%) at each time point is represented in the bar graph above (connected by arrows).

A possible advantage of biotin-prototrophic strains upon contamination of cultures with biotin auxotrophs was investigated by cocultivation of biotin-auxotrophic and biotin-prototrophic S. cerevisiae strains. S. cerevisiae strains IMX585 (*Spycas9*) and IMK827 (*Spycas9 Scbio1*Δ) were equipped with an expression cassette encoding Venus fluorescent protein, yielding strains IMX2212 and IMX2240, respectively. The biotin-prototrophic strain IMX1860 (*Spycas9 CfBIO1↑ Scbio*Δ) was engineered to overexpress the mRuby2 fluorescence protein, yielding strain IMX2238. First, the strains IMX2240 (*Spycas9* Venus↑ *Scbio1*Δ) and IMX2238 (*Spycas9* mRuby2↑ *CfBIO1↑ Scbio*Δ) were mixed in a 40:60 ratio. The relative abundance of the two fluorescent populations was monitored by flow cytometry during consecutive batch cultivation cycles, both in SMG with biotin and in biotin-free SMG. In biotin-containing media, the relative abundance of the two strains remained constant after inoculation (Fig. 6, top). Instead, within 5 h of cultivation in biotin-free medium (Fig. 6, bottom), 75% of the population already consisted of mRuby2-expressing, biotin-prototrophic cells, while during the third and fourth batch cultivation cycles, 99% of the culture consisted of biotin-prototrophic cells. In a similar experiment, the biotin prototroph IMX2238 (*Spycas9* mRuby2↑ *CfBIO1↑ Scbio*Δ) was inoculated together with a fluorescent CEN.PK113-7D-derived strain, IMX2212 (*Spycas9* Venus↑), which carries all native biotin biosynthetic genes (Fig. 7). The initial ratio of 60:40 was maintained in three batches in biotin-containing SMG (Fig. 7, top). In contrast, in biotin-free SMG, the abundance of the auxotroph was reduced to 2% of the total fluorescent population after three batch cultivation cycles (Fig. 7, bottom). These results indicated that while the expression of *CfBIO1* (IMX2238) provided a significant growth advantage in biotin-free SMG, biotin is not released into the medium in amounts sufficient to support the growth of a complete biotin auxotroph or of a strain with a very low biosynthesis capacity, such as CEN.PK113-7D.

### Bio1 proteins show similarity to iron(II)-dependent oxidoreductases, and biotin synthesis requires oxygen.

**FIG 8:**
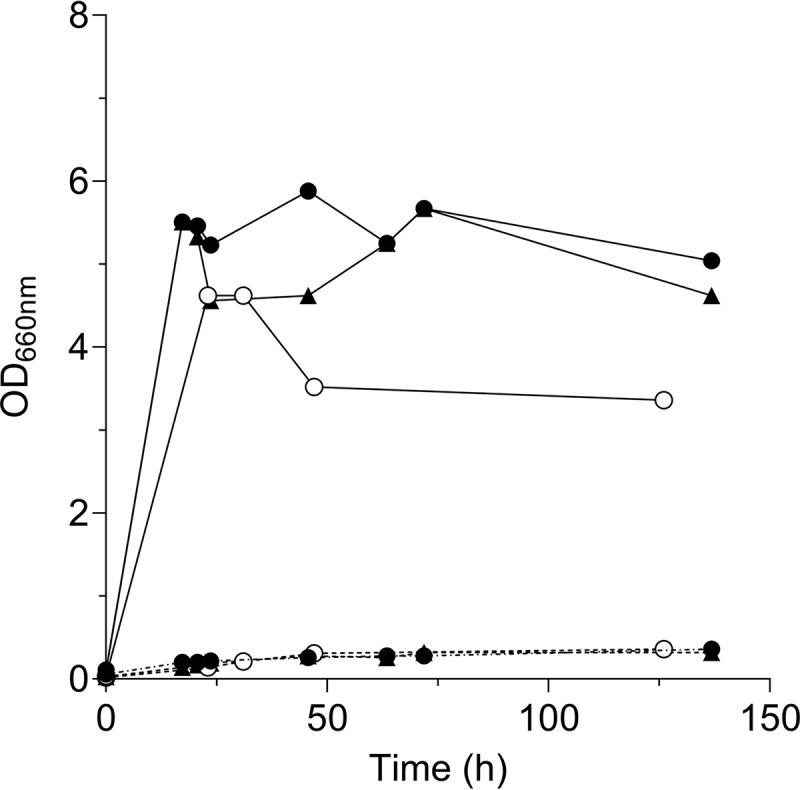
Culture optical density (OD_660_) over time of IMX1859 (*CfBIO1*; black circle), IMS0481 (isolate of biotin prototrophy evolution; white circle), and IMX1511 (*ScBIO1*; black triangle) in SMG (solid line) and SMG without biotin (dashed line) under anaerobic conditions. Specific growth rates were measured after three consecutive transfers. The growth data (OD_660_) presented are single batches representative for aerobic and anaerobic growth profiles of IMX1859, IMS0481, and IMX1511.

For a first exploration of the unknown reaction catalyzed by *Cf*Bio1, its protein structure was predicted by homology modeling with Phyre2. The *Cf*Bio1 structure showed high structural similarity to an Aspergillus nidulans phytanoyl-CoA dioxygenase (PDB entry 5DAQ, EC 1.14.11.18). This oxidoreductase enzyme catalyzes an iron(II)-dependent reaction in which phytanoyl-CoA is oxidized to 2-hydroxyphytanoyl-CoA with 2-oxo-glutarate and oxygen as cosubstrates, yielding succinate and carbon dioxide as by-products (28). The same approach was applied to *Sc*Bio1, which showed high structural similarity to another putative oxygenase (PDB entry 4NAO, EC 1.14.11.18). These two homology hits were retrieved for all the orthologous Bio1 candidate polypeptides except *Yl*Bio1, which instead aligned more optimally with the human phytanoyl-CoA dioxygenase (PDB entry 2OPW, EC 1.14.11.18). In addition to the structural homology to oxidoreductases (EC 1) and more specifically to enzymes using molecular oxygen (EC 1.14), all Bio1 orthologs harbored a conserved 2-oxoglutarate- and iron(II)-dependent oxygenase superfamily domain (pfam03171) (29). These structural similarities strongly suggested that the reaction catalyzed by Bio1 requires oxygen. To test whether growth in biotin-free medium was indeed oxygen dependent, the S. cerevisiae strains IMX1859 (*Spycas9 CfBIO1*↑) and IMS0481 (CEN.PK113-7D), evolved for biotin-independent growth (15), were anaerobically incubated in biotin-free medium. In contrast to their biotin prototrophy under aerobic conditions, neither of the strains was able to grow under anaerobic conditions without biotin supplementation (Fig. 8). These results identify biotin biosynthesis in yeast as oxygen dependent and strongly indicate that the reaction catalyzed by Bio1 requires molecular oxygen.

## DISCUSSION

The elimination of nutritional requirements in industrially relevant microorganisms by the acquisition of new metabolic functions can contribute to process robustness and economics. In this study, we report a novel and simple metabolic engineering strategy to convert biotin-auxotrophic S. cerevisiae strains into biotin prototrophs. The transferable *CfBIO1* expression cassette was identified by a screening of different Saccharomycotina yeasts for their ability to grow in the absence of biotin. Fast-growing, biotin-independent strains from six species belonging to diverse and distant phylogenetic groups were identified (Y. lipolytica from the Yarrowia family, *P. krudiavzevii* from the Pichiaceae family, *W. ciferrii* and *C. fabianii* from the Phaffomycetaceae family, and *L. kluyveri* and *T. delbrueckii* from the Saccharomycetaceae family). Some of these yeasts have already been explored for their biotechnological properties, such as Y. lipolytica for the production of oleochemicals (30), *P. kudriavzevii* as a multistress-tolerant yeast for bioethanol (31) and succinic acid production (32), and *W. ciferrii* for the production of sphingolipids (33). However, their biotin-prototrophic phenotype has not yet been exploited. *C. fabianii* (syn. *Hansenula fabianii*, *Pichia fabianii*, and *Lindnera fabianii*) is not an industrial yeast and has, in rare cases, been isolated from patients with blood infections. Its biotin prototrophy might be related to its opportunistic behavior by providing a selective advantage in low-nutrient environments (34).

Establishing sustainable and cost-effective preparation of growth media remains an important factor in industrial process design and economics (35–37). The benefit of microbes growing independently from the addition of organic growth factors, such as biotin, enables increased standardization as well as reduced costs and time for medium preparation and simplified downstream processing (15, 38). Economic incentives to develop simpler growth media are especially strong for the production of commodity chemicals and in strictly regulated processes that require purified medium components (39). As demonstrated in this study, biotin-independent S. cerevisiae strains did not secrete amounts of biotin in the medium that enabled cross-feeding of fast-growing auxotrophic yeasts. This observation suggests that fermentation processes based on such prototrophs are less susceptible to contamination by biotin-requiring wild S. cerevisiae strains or other biotin-auxotrophic contaminants (40).

The inspection of available draft genome assemblies of the biotin-prototrophic yeast species led to the identification of candidate *BIO1* genes. Surprisingly, only the expression of the *BIO1* ortholog from *C. fabianii* supported fast growth of S. cerevisiae in biotin-free medium. Even the *BIO1* ortholog from *W. ciferrii*, which belongs to the same phylogenetic subdivision of the Saccharomycotina tree as *C. fabianii*, failed to support the growth of S. cerevisiae in the absence of biotin. Without a functional analysis of these *BIO1* orthologs in their original hosts, the possibility remains that these open reading frames do not encode the enzyme performing the initial step in biotin biosynthesis but have a different physiological role. We also cannot exclude differences in posttranslation regulation between the donor organism and S. cerevisiae, which could result in nonfunctional proteins. As shown for the elucidated biotin biosynthetic pathways in prokaryotic model organisms, such as Escherichia coli and B. subtilis, the precursor initiating the biosynthesis could differ. In E. coli, biotin is produced by the so-called BioC-BioH pathway, which diverts malonyl-CoA from fatty acid synthesis by masking the ϖ-carboxyl group of a malonyl-CoA thioester, resulting in the recognition of this uncommon substrate by the E. coli fatty acid synthesis machinery. In two successive rounds of fatty acid synthesis, the malonyl thioester methyl ester is elongated, yielding pimeloyl-acyl carrier protein (ACP) methyl ester, which reversibly esterified to free pimeloyl-ACP, which subsequently enters biotin synthesis (41). In B. subtilis, two distinct pathways have been identified. The indispensable route via the pimeloyl-CoA synthetase BioW (42) activates free pimelic acid with coenzyme A, yielding pimeloyl-CoA (21), which is used by B. subtilis BioF to produce KAPA (43). Alternatively, a second, apparently redundant route (44) via a cytochrome P450-dependent carbon bond-cleaving oxygenase encoded by *bioI* can generate pimeloyl-ACP from long-chain (C_14_, C_16_, or C_18_) acyl-ACP molecules (45). Thus, it cannot be excluded that, similarly, the precursor to produce pimeloyl-ACP or -CoA differs between these yeasts and S. cerevisiae.

While the expression of *CfBIO1* in the NCYC3233 (PE-2) strain resulted in biotin prototrophy, as in any other genetic background tested, the simple integration of this gene at the *SGA1* locus negatively impacted cell growth by reducing by half the strain-specific growth rate, a phenomenon that remains enigmatic. This difference in phenotype might be the result of unwanted mutations introduced during the strain transformation procedure. As previously shown, the induction of aneuploidy is relatively frequent and associated with an aneuploidy-associated stress response (AASR) that may lead to strain growth rate reduction (46, 47). However, the methodology to detect this type of mutation, such as full-genome sequencing of the PE-2-derived transformants, was not used in the framework of this study. This might also be linked to the *SGA1* genetic context, such that integration at that locus in NCYC3233 might have disturbed the expression of surrounding genes. The deregulation of *XBP1*, distal to *SGA1* on CHRIX, might lead to slower growth. *XBP1* is a transcriptional repressor that acts on the promoter of cyclin genes and whose overexpression is known to results in a decreased growth rate (48). Integration at a different chromosomal site would be sufficient to disprove this hypothesis.

Although the amino acid similarities of the six Bio1 orthologs ranged from 15% to 62%, all contained common structural motifs typical for phytanoyl-CoA dioxygenases and 2-oxoglutarate-iron(II)-dependent dioxygenases. These oxidoreductases catalyze reactions that incorporate oxygen from molecular oxygen (O_2_) into their substrates while oxidizing 2-oxoglutarate to succinate and carbon dioxide (49). Consistent with the involvement of an oxygenase in biotin synthesis, our results show that *de novo* biotin biosynthesis represents a previously unidentified oxygen-requiring process in S. cerevisiae and other biotin-prototrophic Saccharomycotina yeasts, in addition to well-known oxygen-requiring processes, such as the synthesis of ergosterol, unsaturated fatty acids (50, 51), and nicotinic acid (52).

An oxygen requirement for biotin synthesis also has been reported for B. subtilis, in which pimeloyl-ACP is generated by the cleavage of a carbon bond of an acyl-ACP (C_14_, C_16_, or C_18_) molecule by the cytochrome P450-dependent, oxygen-requiring enzyme BioI (45). This mechanism provides a direct link between biotin synthesis and fatty acid synthesis. Five of the biotin-prototrophic yeasts identified in this study, Y. lipolytica (53), *W. ciferrii*, *P. kudriavzevii*, *T. delbrueckii*, and *C. fabianii*, are classified as oleaginous yeasts (54), which might support the link between biotin and fatty acid biosynthesis in yeast. Biochemical studies on the reaction(s) catalyzed by *Sc*Bio1 and similar proteins are essential to finally elucidate one of the very few missing reactions in fungal vitamin biosynthesis (55).

## MATERIALS AND METHODS

### Strains, media, and maintenance.

Strains of 35 Saccharomycotina yeasts were tested for growth in the absence of biotin. The S. cerevisiae strains used and constructed in this study belong to the CEN.PK, S288C, Ethanol Red, NCYC 3233/PE-2, and CBS 8066 lineages (Table 2).

**TABLE 2:** Strains used in this study

Strain	Yeast species	Genotype	Description	Reference or source
CBS 5679	Schizosaccharomyces japonicus	Wild type		22
CBS 10395	Schizosaccharomyces pombe	Wild type		22
W29	Yarrowia lipolytica	Wild type		22
CBS 1993	Kuraishia capsulata	Wild type		22
CBS 4732	Ogataea polymorpha	Wild type		22
CBS 11895	Ogataea parapolymorpha	Wild type		22
CBS 2499	Brettanomyces bruxellensis	Wild type		22
CBS 5147	Pichia kudriavzevii	Wild type		22
CBS 2352	Hyphopichia burtonii	Wild type		22
CBS 767	Debaryomyces hansenii	Wild type		22
CBS 6054	Scheffersomyces stipitis	Wild type		22
CBS 7064	Millerozyma farinosa	Wild type		22
CBS 111	Wickerhamomyces ciferrii	Wild type		22
CBS 5481	Cyberlindnera fabianii	Wild type		22
CBS 3082	Lachancea kluyveri	Wild type		22
CBS 6340	Lachancea thermotolerans	Wild type		22
CBS 270.75	Eremothecium cymbalariae	Wild type		22
CBS 6556	Kluyveromyces marxianus	Wild type		22
CBS 2359	Kluyveromyces lactis	Wild type		22
CBS 813	Torulaspora delbrueckii	Wild type		22
CBS 732	Zygosaccharomyces rouxii	Wild type		22
CBS 2163	Vanderwaltozyma polyspora	Wild type		22
CBS 4417	Tetrapisispora phaffii	Wild type		22
CBS 4309	Naumovozyma castellii	Wild type		22
CBS 2517	Kazachstania africana	Wild type		22
CBS 8638	Kazachstania bulderi	Wild type		22
CBS 2170	Nakaseomyces delphensis	Wild type		22
CBS 5792	Saccharomyces bayanus	Wild type		22
CBS 12357	Saccharomyces eubayanus	Wild type		67
CBS 10644	Saccharomyces arboricolus	Wild type		22
CBS 8840	Saccharomyces kudriavzevii	Wild type		22
CBS 8839	Saccharomyces mikatae	Wild type		22
CBS 1190	Saccharomyces paradoxus	Wild type		22
CBS 6412	Saccharomyces cerevisiae	Wild type	Kyokai no. 7	22
CEN.PK113-7D	Saccharomyces cerevisiae	*MAT***a** *MAL2-8c SUC2*	Laboratory strain	68
S288C	Saccharomyces cerevisiae	*MAT*α *SUC2 gal2 mal mel flo1 flo8-1 hap1 ho bio1 bio6*	Laboratory strain	69
CBS 8066	Saccharomyces cerevisiae	*MAT***a***/α HO/ho*	Laboratory strain	22
Ethanol Red	Saccharomyces cerevisiae	*MAT***a***/α*	Industrial bioethanol production strain	F. R. Lesaffre
NCYC 3233	Saccharomyces cerevisiae	*MAT***a***/α*	Brazilian bioethanol production strain PE-2	70
IMS0481	Saccharomyces cerevisiae	*MAT***a** evolved	Evolved CEN.PK113-7D for full biotin protrophy	15
IMX585	Saccharomyces cerevisiae	*MAT***a** *can1*Δ::*cas9-natNT2*	CEN.PK113-7D expressing *Spycas9*	26
IMX1511	Saccharomyces cerevisiae	*MAT***a** *can1*Δ::*cas9-natNT2 sga1*Δ::*ScPYK1*p-*ScBIO1-ScBIO1*t ScBIO1t	*ScBIO1↑*	This study
IMX1862	Saccharomyces cerevisiae	*MAT***a** *can1*Δ::*cas9-natNT2 sga1*Δ::*ScPYK1*p-*YlBIO1-ScBIO1*t	*YlBIO1↑*	This study
IMX1861	Saccharomyces cerevisiae	*MAT***a** *can1*Δ::*cas9-natNT2 sga1*Δ::*ScPYK1*p-*PkBIO1-ScBIO1*t	*PkBIO1↑*	This study
IMX1863	Saccharomyces cerevisiae	*MAT***a** *can1*Δ::*cas9-natNT2 sga1*Δ::*ScPYK1*p-*WcBIO1-ScBIO1*t	*WcBIO1↑*	This study
IMX1859	Saccharomyces cerevisiae	*MAT***a** *can1*Δ::*cas9-natNT2 sga1*Δ::*ScPYK1*p-*CfBIO1-ScBIO1*t	*CfBIO1↑*	This study
IMX1857	Saccharomyces cerevisiae	*MAT***a** *can1*Δ::*cas9-natNT2 sga1*Δ::*ScPYK1*p-*TdBIO1-ScBIO1*t	*TdBIO1↑*	This study
IMX1858	Saccharomyces cerevisiae	*MAT***a** *can1*Δ::*cas9-natNT2 sga1*Δ::*ScPYK1*p-*LkBIO1-ScBIO1*t	*LkBIO1↑*	This study
IMX1860	Saccharomyces cerevisiae	*MAT***a** *can1*Δ::*cas9-natNT2 Scbio1*Δ *sga1*Δ::*ScPYK1*p-*CfBIO1-ScBIO1*t	*Scbio1*Δ *CfBIO1↑*	This study
IMK827	Saccharomyces cerevisiae	*MAT***a** *can1*Δ::*cas9-natNT2 Scbio1*Δ	Biotin auxotroph	This study
IMX2212	Saccharomyces cerevisiae	*MAT***a** *can1*Δ::*cas9-natNT2 x-2*Δ:: *ScTEF1*p-Venus-*ScTDH1*t	Venus*↑* biotin auxotroph	This study
IMX2238	Saccharomyces cerevisiae	*MAT***a** *can1*Δ::*cas9-natNT2 Scbio1*Δ *sga1*Δ::*ScPYK1*p-*CfBIO1-ScBIO1*t *x-2*Δ::*ScPGK1*p-mRuby2-*ScPGK1*t	mRuby2*↑* biotin prototroph	This study
IMX2240	Saccharomyces cerevisiae	*MAT***a** *can1*Δ::*cas9-natNT2 Scbio1*Δ *x-2*Δ::*ScTEF1*p-Venus-*ScTDH1*t	Venus*↑* biotin auxotroph	This study
IMX2103	Saccharomyces cerevisiae	*sga1*Δ::*ScPYK1*p-*CfBIO1-ScBIO1*t, *ScPGK1*p-*ScBIO6-ScBIO6*t	S288C *CfBIO1*↑ *ScBIO6*↑	This study
IMX2104	Saccharomyces cerevisiae	*sga1*Δ::*ScPYK1*p-*CfBIO1-ScBIO1*t	CBS 8066 *CfBIO1*↑	This study
IMX2101	Saccharomyces cerevisiae	*sga1*Δ::*ScPYK1*p-*CfBIO1-ScBIO1*t	Ethanol Red *CfBIO1*↑	This study
IMX2090	Saccharomyces cerevisiae	*sga1*Δ::*ScPYK1*p-*CfBIO1-ScBIO1*t	NCYC 3233 *CfBIO1*↑	This study

Yeast strains were grown on YP medium (10 g liter^−1^ yeast extract, 20 g liter^−1^ peptone) or on synthetic medium (SM) containing 3.0 g liter^−1^ KH_2_PO_4_, 5.0 g liter^−1^ (NH_4_)_2_SO_4_, 0.5 g liter^−1^ MgSO_4_, 7·H_2_O, 1 ml liter^−1^ trace element solution (4.5 mg liter^−1^ ZnSO_4_·7H_2_O, 0.3 mg liter^−1^ CoCl_2_·6H_2_O, 1 mg liter^−1^ MnCl_2_·4H_2_O, 0.3 mg liter^−1^ CuSO_4_·5H_2_O, 4.5 mg liter^−1^ CaCl_2_·2H_2_O, 3 mg liter^−1^ FeSO_4_·7H_2_O, 0.4 mg liter^−1^ NaMoO_4_·2H_2_O, 1 mg liter^−1^ H_3_BO_3_, 0.1 mg liter^−1^ KI, 15 mg liter^−1^ EDTA), and 1 ml liter^−1^ vitamin solution [0.05 g liter^−1^ d-(+)-biotin, 1.0 g liter^−1^ d-calcium pantothenate, 1.0 g liter^−1^ nicotinic acid, 25 g liter^−1^ *myo*-inositol, 1.0 g liter^−1^ thiamine hydrochloride, 1.0 g liter^−1^ pyridoxol hydrochloride, 0.2 g liter^−1^ 4-aminobenzoic acid] (56). The pH was adjusted to 6 with 2 M KOH prior to autoclaving at 120°C for 20 min. Vitamin solutions were sterilized by filtration and added to the sterile medium. Concentrated sugar solutions were autoclaved at 110°C for 20 min and added to the sterile medium to give a final concentration of 20 g liter^−1^ glucose (yeast extract-peptone-dextrose [YPD] and SMG). Biotin-free SM was prepared similarly, but biotin was omitted from the vitamin solution. Similarly, after autoclaving concentrated glucose solution at 110°C for 20 min, glucose was added to biotin-free SM to a final concentration of 20 g liter^−1^ (biotin-free SMG). Solid medium contained 1.5% Bacto agar and, when indicated, acetamide for SMG acetamide (20 g liter^−1^ glucose, 1.2 g liter^−1^ acetamide, 3.0 g liter^−1^ KH_2_PO_4_, 6.6 g liter^−1^ K_2_SO_4_, 0.5 g liter^−1^ MgSO_4_ 7·H_2_O, 1 ml liter^−1^ trace element solution, and 1 ml liter^−1^ vitamin solution) and 200 mg liter^−1^ hygromycin for YPD hygromycin.

E. coli cells (XL1-Blue; Agilent Technologies, Santa Clara, CA) were grown in lysogeny broth (LB) medium (5.0 g liter^−1^ yeast extract, 10 g liter^−1^ Bacto tryptone, 5.0 g liter^−1^ NaCl) supplemented with 25 mg liter^−1^ chloramphenicol, 100 mg liter^−1^ ampicillin, or 50 mg liter^−1^ kanamycin. Solid LB medium contained 2.0% Bacto agar. For maintenance, stock cultures of yeast strains were grown on YPD, if not specified differently, and E. coli cultures on LB medium with an appropriate antibiotic marker until late exponential phase, complemented with sterile glycerol to a final concentration of 30% (vol/vol), and stored at −80°C as 1-ml aliquots until further use.

### Shake flask cultivation conditions.

Cultivation experiments for the determination of biotin requirements of yeasts were performed as follows. A 1-ml aliquot of a stock culture was inoculated in 100 ml SMG in a 500-ml shake flask and incubated for 20 h at 30°C. A second 100-ml SMG culture was started by inoculating 2 ml of the first shake flask culture. When the second culture reached mid-exponential phase (optical density at 660 nm [OD_660_] of 3 to 5), it was used to inoculate a third culture at an OD_660_ of 0.1 to 0.3. Similarly, a 1-ml aliquot of a thawed stock culture was inoculated in 100 ml biotin-free SMG in a 500-ml shake flask and incubated for 20 h at 30°C. A second 100-ml biotin-free SMG culture was started by inoculating 2 ml of the first shake flask culture. If the second culture reached mid-exponential phase (OD_660_ of 3 to 5), it was used to inoculate a third culture at an OD_660_ of 0.1 to 0.3. Shake flasks were incubated as biological duplicates at 30°C and 200 rpm in an Innova incubator (Brunswick Scientific, Edison, NJ). Strains IMX585 and CEN.PK113-7D, which consistently failed to grow on biotin-free SMG in the third culture, were used as a negative control in all growth experiments.

Growth of cultures was monitored by OD_660_ of an appropriate dilution of the third shake flask culture, measured with a Jenway 7200 Spectrophotometer (Cole-Palmer, Stone, United Kingdom). Specific growth rates were calculated from a minimum of six data points during exponential growth, covering 3 to 4 doublings of the OD_660_. Growth rate was calculated using the equation *X* = *X*_0_*e*^μ^*^t^*, in which μ indicates the exponential growth rate. All aerobic shake flask experiments were carried out in an Innova shaker incubator (New Brunswick Scientific, Edison, NJ) set at 30°C and 200 rpm.

For growth profiling under anaerobic conditions, first and second cultures were grown in 40 ml SMG or biotin-free SMG in a 50-ml shake flask, as described previously, under aerobic conditions. Two hundred-microliter samples of mid-exponentially growing cells from the second culture were transferred to an anaerobic workstation (Bactron; Sheldon Manufacturing, Cornelius, OR) at 30°C and used to inoculate the third culture. Oxygen entry through the airlock of the anaerobic workstation was minimized with the use of a regenerated Pd catalyst for H_2_-dependent oxygen removal that was introduced into the chamber. Concentrated solutions of Tween 80 and ergosterol were added to the medium, aiming for final concentrations of 420 mg liter^−1^ and 10 mg liter^−1^, respectively. To deplete all of the nutrients from the aerobic growth phase, a fourth culture was inoculated from exponentially growing cells. The OD_660_ of the fourth culture was measured with an Ultrospec 10 cell density meter (Biochrom; Harvard Bioscience, Cambridge, United Kingdom), which was placed inside the anaerobic workstation. All anaerobic experiments were carried out in biological duplicates as described before (57).

In the case of cocultivations, the strains were inoculated in 100 ml SMG and biotin-free SMG in a 500-ml shake flask by addition of a 1-ml aliquot of a stock culture and incubated for 20 h at 30°C. A second 100-ml SMG culture was started by inoculating 2 ml of the first shake flask culture. When the second culture reached mid-exponential phase (OD_660_ of 3 to 5), it was used to inoculate a third culture at an OD_660_ of 0.1 to 0.3 and an approximate 60:40 mix of the two strains from the same medium (biotin supplemented or biotin free). After reaching late exponential phase, cocultures were transferred into fresh medium to an OD_660_ of 0.2. The fifth and sixth consecutive cultures were started similarly by transferring stationary-phase cultures from the previous batch. During the third, fourth, fifth, and sixth cultures, samples were taken to analyze the OD_660_ and population distribution by flow cytometry.

### Molecular biology techniques.

DNA fragments were amplified by PCR amplification with Phusion Hot Start II high-fidelity polymerase (Thermo Fisher Scientific, Landsmeer, Netherlands) and desalted or PAGE-purified oligonucleotide primers (Sigma-Aldrich, St. Louis, MO) (Table 3) according to the manufacturers’ instructions. For diagnostic PCR analysis of constructed plasmids and strains, DreamTaq polymerase (Thermo Fisher Scientific) was used according to the manufacturer’s recommendations. PCR products were separated by electrophoresis on 1% (wt/vol) agarose gels in Tris-acetate-EDTA (TAE) buffer (Thermo Fisher Scientific) with SERVA DNA stain G (1:100,000) (Serva, Heidelberg, Germany) and, if required, purified with a Zymoclean gel DNA recovery kit (Zymo Research, Irvine, CA) or GenElute PCR clean-up kit (Sigma-Aldrich). Yeast strains of the CEN.PK lineage, S288C, and NCYC 3233/PE-2 were transformed by the lithium acetate (LiAc) method. Yeast strains Ethanol Red and CBS 8066 were transformed using electroporation, as previously described (58), with a 2-mm cuvette (Bio-Rad, Hercules, CA) using a Gene Pulser XCell electroporation system (Bio-Rad). Yeast genomic DNA was isolated using the YeaStar genomic DNA kit (Zymo Research) or using the SDS-LiAc protocol (59). E. coli cells were chemically transformed and plated on selective LB agar medium. Plasmids from selected clones were isolated from E. coli with a Sigma GenElute plasmid kit (Sigma-Aldrich).

**TABLE 3:** Primers used in this study

Primer no.	Sequence 5′→3′
3841	CACCTTTCGAGAGGACGATG
4892	TACAGACACGACGCATGG
5328	ATTTTAGCGTAAAGGATGGG
5941	GCTGGCCTTTTGCTCACATG
6005	GATCATTTATCTTTCACTGCGGAGAAG
6006	GTTTTAGAGCTAGAAATAGCAAGTTAAAATAAGGCTAGTC
7428	TGTGATGATGTTTTATTTGTTTTGATTGG
7469	GGAGTTGACCGTCTTAACAG
8737	ATAACGGGTTTCTCGCTGAA
10235	TTGTGCGGTTTCGTTTAGGG
10320	CATGCGCGGATGACACGAAC
10325	AGTCATCCGAGCGTGTATTG
10873	ACGTGCGGAATAGGAATCTC
11037	AGTTCTTTGTAGAACAGCTCTC
11614	GCATCGTCTCATCGGTCTCATATGATGAACACAAAATCACTCG
11615	ATGCCGTCTCAGGTCTCAGGATTTACTCTTTATCGTCATAAATAATCTTG
11618	GCATCGTCTCATCGGTCTCAATCCATATCATCATTTCTGCCACAAATATATG
11619	ATGCCGTCTCAGGTCTCACAGCCGGTAGCTTGACGTGCGGAATAG
11898	CGCGGAAACGGGTATTAGGG
11899	CTAGATCCGGTAAGCGACAG
11915	GAGTGAGTGCTTTGTTCAATGG
11945	AGCATCACCTTCACCTTCAC
12086	TTTACAATATAGTGATAATCGTGGACTAGAGCAAGATTTCAAATAAGTAACAGCAGCAAAGCCGATAATTGCAGACGAAC
12108	TAAATATCTAGGAAATACACTTGTGTATACTTCTCGCTTTCCTTTTATTTTTTTTTTTGTCATGCTCAGCCGGTAGCTTG
12223	CCAGGTGGCGTGCTAAACTTTTATAATGTATAAAAACCACCACCTCATAAAGTTTACTGGATATCATCATTTCTGCCACAAATATATGTACTGAGTCTATACGTCAAAGTAAAAAAATAA
12224	TTATTTTTTTACTTTGACGTATAGACTCAGTACATATATTTGTGGCAGAAATGATGATATCCAGTAAACTTTATGAGGTGGTGGTTTTTATACATTATAAAAGTTTAGCACGCCACCTGG
12616	CGAGTCAGTGAGCGAGGAAG
12991	GCATCGTCTCATCGGTCTCATATGGCGTCTAAGAACCAAAA
12992	ATGCCGTCTCAGGTCTCAGGATCTACTCAAAAACTGCATGGA
13038	GCATCGTCTCATCGGTCTCATATGACGTTTAGTAAGAACTTAAC
13039	ATGCCGTCTCAGGTCTCAGGATCTATTCGATAACAGGATAAA
13287	AACAAGATCCGAGTACTTAG
13290	TTCGTCTCTCTCACACTTATACGGGTCGTTAGT
13291	CACGTCTCATGAGCCCGGAATAAATCTAGTGGC
13293	ATATCGTCCCTAGTCAATTC
13596	GCTGAAGATTTATCATACTATTCCTCCGCTCGTTTCTTTTTTCAGTGAGGTGTGTCGTGATGAACTGGCCGATAATTGCAGA
13597	ATTCTCGCCAAGGCATTACCATCCCATGTAAGAACGGAATAAAACAGCATTCGAAGGTTATGATGACCCCGTCGTCTCATT
13662	TCCTCGGGCAGAGAAACTCG
13963	CAAATAAAACATCATCACATATGACACATATAAGTAACTCGGAGTATC
13964	GCAGAAATGATGATATGGATTTATATACGAACTACTTTACAAACATCA
14139	TGCGCATGTTTCGGCGTTCGAAACTTCTCCGCAGTGAAAGATAAATGATCAGGAAACGCTACTCGGAGTAGTTTTAGAGCTAGAAATAGCAAGTTAAAATAAG
14162	GGCACCTCTGGCTTGTCTTC
14167	TACTCCGAGTAGCGTTTCCT
14661	TTAGGGAGCACATCCATGCCAATAGCTCGACAAGCGGCGAGAGCCTTGCACCTATGCTATCAATAGGCACACTGTAATTG
14662	GTATATATATATATTTGATGTAAATATCTAGGAAATACACTTGTGTATACTTCTCGCTTTCTAAACTGAGCACAAGTTTC
14663	ATAGCATAGGTGCAAGGCTCTCGCCGCTTGTCGAGCTATTGGCATGGATGTGCTCCCTAATAGCTTGACGTGCGGAATAG
14891	CATATCATCATTTCTGCCACAAATAT
14892	CAATCAAAACAAATAAAACATCATCACAATGAGTTTCAACTACGAAGACTG
14893	ATATTTGTGGCAGAAATGATGATATGTTAGTCGTATAAAACTTTACATACTT
14907	AATGCAATGGAGCTTGAGAC
14909	TGGTCGTCTCTATGCAAAGG
14925	GCATCGTCTCATCGGTCTCATATGTGCTGTACATGTACATACCA
14926	ATGCCGTCTCAGGTCTCAGGATTAGGCGCGTAAAGTTATTAC
14928	GGGAGGTCGCAATATCTCTG
15104	GCAGAAATGATGATATGGATTTAATTAACATCTGAGACTT
15105	CAAATAAAACATCATCACATATGACTGTTATAGATACCAATGA
16792	TCACAGAGGGATCCCGTTACCCATCTATGCTGAAGATTTATCATACTATTCCTCCGCTCGGCCTTGCCAACAGGGAGTTC
16793	GTCATAACTCAATTTGCCTATTTCTTACGGCTTCTCATAAAACGTCCCACACTATTCAGGCGTTCAGGGTAATATATTTT

### Plasmid construction.

**(i) Construction of part plasmids using yeast toolkit principles.** Coding sequences of putative *ScBIO1* orthologs from Y. lipolytica W29, *P. kudriavzevii* CBS 5147, *W. ciferrii* CBS 111, *C. fabianii* CBS 5481, *L. kluyveri* CBS 3082, and *T. delbrueckii* CBS 813 were obtained by PCR with primer combinations 14925/14926, 14892/14893, 15104/15105, 13963/13964, 13291/13039, 13290/13038, and 12991/12992 using genomic DNA of the respective yeast as a template. In the case of S. cerevisiae CEN.PK113-7D *BIO1*, the plasmid pUDE450 (Table 4) (15) was isolated from E. coli cultures and used as a template for PCR with primer pair 11614/11615. The *ScBIO1* terminator (*ScBIO1*t) was similarly PCR amplified using primer pair 11618/11619 and plasmid pUDE450 as the template. The DNA fragments containing *BIO1* coding sequences from Y. lipolytica, *T. delbrueckii*, *L. kluyveri*, and CEN.PK113-7D, as well as *ScBIO1*t, were *in vitro* assembled with entry vector pUD565 using BsmBI-T4 ligase-directed Golden Gate cloning (60), resulting in yeast toolkit type 3 plasmids pGGkp243, pGGkp169, pGGkp178, and pGGkp080, respectively, and a yeast toolkit type 4 plasmid with *ScBIO1*t pGGkp078 (Table 4). To remove a BsaI restriction recognition site in the coding sequence of *LkBIO1*, the open reading frame was PCR amplified from *L. kluyveri* CBS 3082 genomic DNA using two primer pairs, 13291/13039 and 13290/13038, with overhangs allowing for BsmBI-T4 ligase-directed Golden Gate cloning of the two DNA fragments into entry vector pUD565 (61), leaving behind a sequence without a BsaI site and resulting in the yeast toolkit type 3 plasmid pGGkp178. After *in vitro* assembly, plasmids were transformed into E. coli and plated on LB chloramphenicol for selection. The yeast toolkit type plasmids pGGkp080, pGGkp169 pGGkp078, and pGGkp178 were confirmed by diagnostic PCR with primer pair 12616/4892, 12616/13287, 12616/10235, and 12616/13290, respectively. Yeast toolkit type plasmid pGGkp243 was confirmed by restriction analysis with restriction enzymes PvuII and DraI. The promoter *ScPYK1*p was synthesized by GeneArt (Thermo Fisher Scientific) and cloned as plasmid pGGkp117. The yeast toolkit type plasmids were stored in transformed E. coli cultures.

**TABLE 4:** Plasmids used in this study

Name	Characteristic(s)	Reference or source
pGGkd005	*hph CEN6/ARS4 bla* ColE1 Gfp dropout	This study
pGGkd015	*bla* ColE1 Gfp dropout	61
pGGkd017	*URA3* 2μ bla ColE1 Gfp dropout	This study
pGGKp078	*cat ScBIO1*t	This study
pGGKp080	*cat ScBIO1*	This study
pGGkp117	*cat ScPYK1*p	This study
pGGkp169	*cat TdBIO1*	This study
pGGkp178	*cat LkBIO1*	This study
pGGkp243	*cat YlBIO1*	This study
pROS11	*amdS* 2μ gRNA*_CAN1_* gRNA*_ADE2_*	26
pUD565	*cat* Gfp dropout	61
pUD788	*bla* ColE1 *ScPYK1*p-*TdBIO1-ScBIO1*t	This study
pUD789	*bla* ColE1 *ScPYK1*p-*LkBIO1-ScBIO1*t	This study
pUD790	*URA3* 2μ *bla* ColE1 *ScPYK1*p-*CfBIO1-ScBIO1*t	This study
pUD988	*URA3* 2μ *bla* ColE1 *ScPYK1*p-*PkBIO1-ScBIO1*t	This study
pUD989	*bla* ColE1 *ScPYK1*p-*YlBIO1-ScBIO1*t	This study
pUD990	*URA3* 2μ *bla* ColE1 *ScPYK1*p-Wc*BIO1*-Sc*BIO1*t	This study
pUDC193	*URA3 CEN6/ARS4 bla* ColE1 *ScTEF1*p-Venus-*ScTDH1*t	This study
pUDE448	*URA3* 2μ *bla* ColE1 *ScPGK1*p-*ScBIO6-ScBIO6*t	15
pUDE450	*URA3* 2μ *bla* ColE1 *ScPYK1*p-*ScBIO1-ScBIO1*t	15
pUDE480	*hph CEN6/ARS4 bla* ColE1 *ScPGK1*p-mRuby2-*ScPGK1*t	This study
pUDE718	*URA3* 2μ *bla* ColE1 *ScPYK1*p-*ScBIO1-ScBIO1*t	This study
pUDP002	*hph* panARS(OPT) *bla* ColE1 *ScTDH3*p-BsaI sit*e-ScCYC1*t *AaTEF1*p-*Spcas9*^D147Y P411T^-*ScPHO5*t	63
pUDP145	*hph* panARS(OPT) *bla* ColE1 *ScTDH3*p-HH-gRNA*_SGA1_*-HDV-*ScCYC1*t	This study
pUDR119	*amdS* 2μ *ScSNR52*p-gRNA*_SGA1_-SUP4*t	26
pUDR244	*amdS* 2μ *ScSNR52*p-gRNA*_BIO1_-SUP4*t *ScSNR52*p-gRNA*_BIO1_-SUP4*t	This study
pUDR376	*amdS* 2μ *bla* ColE1 *ScSNR52*p-gRNA_X-2_-*SUP4*t	This study
pYTK002	*cat* ConLS	60
pYTK011	*cat ScPGK1*p	60
pYTK046	*cat* mRuby2	60
pYTK047	*cat* Gfp dropout	60
pYTK054	*cat ScPGK1*t	60
pYTK067	*cat* ConR1	60
pYTK072	*cat* ConRE	60
pYTK074	*cat URA3*	60
pYTK079	*cat hph*	60
pYTK081	*cat CEN6/ARS4*	60
pYTK082	*cat* 2μ	60
pYTK083	*bla* ColE1	60
pYTK095	*bla* ColE1 Gfp dropout	60

**(ii) Construction of transcriptional modules.** The control S. cerevisiae CEN.PK113-7D *BIO1* transcriptional module was constructed by Golden Gate cloning combining DNA fragments with compatible overhangs from plasmids pGGkd017, pGGkp117, pGGkp080, and pGGkp078, yielding plasmid pUDE718. The entry plasmid pGGkd017 was also constructed by Golden Gate cloning combining DNA fragments with compatible overhangs from pYTK002, pYTK047, pYTK072, pYTK074, pYTK082, and pYTK083 (60). The *T. delbrueckii BIO1* transcriptional module was constructed by Golden Gate cloning, combining DNA fragments with compatible overhangs from plasmids pGGkd015, pGGkp117, pGGkp169, and pGGkp078, yielding plasmid pUD788. The entry plasmid pGGkd015 was obtained by Golden Gate cloning with yeast toolkit type plasmids pYTK002, pYTK047, pYTK067, and pYTK095. The *L. kluyveri BIO1* transcriptional module was constructed by Golden Gate cloning, combining DNA fragments with compatible overhangs from plasmids pGGkd015, pGGkp117, pGGkp178, and pGGkp078, yielding plasmid pUD789. The Y. lipolytica *BIO1* transcriptional module was constructed by Golden Gate cloning, combining DNA fragment with compatible overhangs from plasmids pGGkd015, pGGkp117, pGGkp243, and pGGkp078, yielding plasmid pUD989. The transcriptional modules of *P. kudriavzevii*, *W. ciferrii*, and *C. fabianii BIO1* genes were constructed by Gibson assembly (62) (New England Biolabs, Ipswich, MA) using pUDE718 as the plasmid backbone, which includes the *ScPYK1*p and *ScBIO1*t sequences. The *BIO1* genes from *P. kudriavzevii* CBS 5147, *W. ciferrii* CBS 111, and *C. fabianii* CBS 5481 were amplified by primer pairs 14892/14893, 15104/15105, and 13963/13964, respectively, using genomic DNA of the respective yeast as a template. DNA fragments were assembled with linearized pUDE718 backbone using primer pair 7428/14891, yielding plasmids pUD988, pUD990, and pUD790, respectively. The assembly mixes were transformed into E. coli and plated on LB-ampicillin for selection. The transcriptional module plasmids were confirmed by diagnostic PCR with the forward primer 10320 and the following gene-specific reverse primers: 13287 for *TdBIO1*, 13293 for *LkBIO1*, 14928 for *YlBIO1*, 4892 for *ScBIO1*, 14909 for *PkBIO1*, 14907 for *WcBIO1*, and 14162 for *CfBIO1*. The transcriptional module for the expression of fluorophore mRuby2 was constructed by Golden Gate cloning, combining DNA fragments with compatible overhangs from plasmids pGGKd005, pYTK011, pYTK046, and pYTK054. The entry plasmid pGGkd005 was constructed by Golden Gate cloning, combining DNA fragments with compatible overhangs from pYTK002, pYTK047, pYTK067, pYTK079, pYTK083, and pYTK081. The assembly mixes were transformed into E. coli and plated on LB ampicillin for selection. The mRuby2 transcriptional module plasmid was confirmed by diagnostic PCR with primer pair 10320/10325 and stored as pUDE480 in transformed E. coli cultures.

**(iii) *Spycas9*-expressing plasmids.** The gRNA*_ScSGA1_*-expressing plasmid pUDR244 was constructed by *in vitro* Gibson assembly. The linearized pROS11 plasmid, obtained by PCR with 6005/6006, was assembled together with a PCR-amplified fragment using primer 14139 and pROS11 as a template as previously described (26). The plasmid DNA was isolated from E. coli and the correct assembly of plasmid pUDR244 confirmed by diagnostic PCR with primers 3841/14167/5941. Similarly, plasmid pUDR376 was assembled with linearized pROS11 and the PCR-amplified DNA fragment but using primer 10866. Assembly of pUDP145 was performed *in vitro* by BsaI-T4 DNA ligase-directed Golden Gate cloning with the gRNA entry plasmid pUDP002 (63) and a *de novo*-synthesized DNA fragment (GeneArt, Thermo Fisher Scientific) encoding a gRNA targeting *ScSGA1*.

### Strain construction.

The *BIO1* transcriptional modules were PCR amplified by using primer pair 12086/12108, adding specific sequences for homologous recombination into the *SGA1* locus in S. cerevisiae, directed by CRISPR/Cas9 (26). The transcriptional module was amplified from plasmid pUD788 for *TdBIO1*, from plasmid pUD789 for *LkBIO1*, from plasmid pUD989 for *YlBIO1*, from plasmid pUDE718 for *ScBIO1*, from plasmid pUD988 for *PkBIO1*, from plasmid pUD990 for *WcBIO1*, and from plasmid pUD790 for *CfBIO1*. Targeting at the *ScSGA1* locus in IMX585 was directed by CRISPR/Cas9 and a target-specific gRNA-expressing plasmid (26). Each transcriptional module was cotransformed with plasmid pUDR119 expressing the gRNA to target Cas9 to *ScSGA1* in strain IMX585 using the LiAc transformation protocol. The transformed cells were plated on selective SMG acetamide and incubated for 3 days at 30°C. Genomic DNA of transformants was isolated using the SDS-LiAc protocol (59). The desired genotype was confirmed using primer pair 11898/11899 and a gene-specific primer pair with 11898 as the forward primer and the following reverse primers: 13287 for *TdBIO1*, 13293 for *LkBIO1*, 14928 for *YlBIO1*, 4892 for *ScBIO1*, 14909 for *PkBIO1*, 14907 for *WcBIO1*, and 14162 for *CfBIO1*. The correct clone was inoculated in 20 ml nonselective YPD for plasmid removal and incubated for 24 h at 30°C. The cells were plated on YPD agar to obtain single-colony isolates. One isolate was restreaked on both SMG acetamide and YPD. When no growth was observed on SMG acetamide, the respective clone was once again confirmed by PCR with gene-specific primers. Furthermore, the genetic modification at the *ScSGA1* locus was verified by Sanger sequencing (BaseClear, Leiden, Netherlands), using primers 11898/11899 to PCR amplify the modified locus and further using primers 11898, 11915, and 10235 for sequencing. The strain with the transcriptional module coding for *TdBIO1* was stocked as IMX1857, *LkBIO1* as IMX1858, *YlBIO1* as IMX1862, *ScBIO1* as IMX1511, *PkBIO1* as IMX1861, *WcBIO1* as IMX1863, and *CfBIO1* as IMX1859 in SMG.

The deletion of the native *ScBIO1* locus in strains IMX1859 and IMX585 was directed by CRISPR/Cas9 using plasmid pUDR244 (Table 4), which was cotransformed with annealed repair oligonucleotides 12223/12224 in strains IMX1859 and IMX585 using the LiAc transformation protocol with SMG acetamide as the selection marker, and the deletion was confirmed using primer pair 7469/10873.

To achieve the integration of the *CfBIO1* module at the *SGA1* locus of CBS 8066, NCYC 3233/PE-2, and Ethanol Red, the plasmid pUDP145 was cotransformed with a PCR-amplified DNA fragment using primer pair 12086/12108 and pUD790 as the template. In contrast to CBS 8066, NCYC 3233/PE-2, and Ethanol Red, the S. cerevisiae strain S288C is missing both the *ScBIO1* and *ScBIO6* genes (27); therefore, the *CfBIO1* transcriptional module was amplified using primer pair 12086/14663 from pUD790, and an additional transcriptional module harboring *ScBIO6* was amplified using primer pair 14661/14662 and plasmid pUDE448 as the template. These two DNA fragments harbored homologous flanks, allowing for *in vivo* assembly into the *ScSGA1* locus after cotransformation with pUDP145. Transformants selected on YPD hygromycin were tested for the desired genotype using primer pair 11898/11899 and using a *CfBIO1*-specific PCR with primer pair 11898/14162. In the case of transformation into strain S288C, an additional diagnostic PCR with primer pair 8737/11899 was conducted. After counterselection, the strain with the *CfBIO1* and *ScBIO6* transcriptional module in S288C was stored as IMX2103. CBS 8066 expressing *CfBIO1* was stored as IMX2104, Ethanol Red expressing *CfBIO1* as IMX2101, and NCYC 3233/PE-2 expressing *CfBIO1* as IMX2090 in SMG.

The Venus fluorophore transcriptional module was PCR amplified from pUDC193 by using primer pair 16792/16793. The mRuby2 fluorophore transcriptional module was PCR amplified from plasmid pUDE480 with primer pair 13596/13597. These two linear DNA fragments contained homologous flanks to the intergenic region X-2 (64) to enable integration at this site when cotransformed with pUDR376 into a Cas9-expressing strain background. The Venus fluorophore gene was integrated into IMX585 and IMK827 and the mRuby2 fluorophore into IMX1860. Genomic DNA of transformants selected on SMG acetamide was isolated using the SDS-LiAc protocol. The desired genotype was confirmed by PCR using primer pair 13662/11037 and a gene-specific primer pair with 13662 as forward primer and 5328 as reverse primer to confirm mRuby2 expression cassette integration. The primer pair 13662/11945 was used to confirm the integration of the Venus expression cassette at the X-2 intergenic site. IMK827 with Venus fluorescence was stored as IMX2240, IMX585 with Venus fluorescence was stored as IMX2212, and IMX1860 with mRuby2 fluorescence was stored as IMX2238 in SMG.

### Flow-cytometric analysis.

Samples from aerobic 100-ml cultures in 500-ml shake flasks were vortexed thoroughly to disrupt cell aggregates and used for flow cytometry on a BD FACSAria II SORP cell sorter (BD Biosciences, Franklin Lakes, NJ) equipped with 355-, 445-, 488-, 561-, and 640-nm lasers and a 70-μm nozzle and operated with filtered FACSFlow (BD Biosciences). Cytometer performance was evaluated prior to each experiment by running a CST cycle with CS&T beads (BD Biosciences). The fluorophore mRuby2 was excited by the 561-nm laser, and emission was detected through a 582-nm bandpass filter with a bandwidth of 15 nm. The fluorophore Venus was excited by the 488-nm laser, and emission was detected through a 545-nm bandpass filter with a bandwidth of 30 nm. For each sample, 10,000 events were analyzed, and the same gating strategy was applied to all samples from the same culture. The reference sample for no fluorescent cells was a mid-exponentially growing culture of IMX585 on SMG. The Venus and mRuby2 fluorescence reference was obtained from mid-exponential aerobic cultures on SMG of IMX2240 or IMX2212 and IMX2238, respectively. Cells without fluorescence and doublets or with Venus and mRuby2 fluorescence were selected in a Venus/mRuby2 plot.

### Sequence analysis and structural modeling.

Genomic DNA of Y. lipolytica W29 was sequenced in-house on a MiSeq sequencer (Illumina, San Diego, CA) to obtain a 300-cycle paired-end library with a fragment length of 550 bp using a PCR-free library preparation, yielding 4.28 million reads with a total sequence of 1.27 gigabases. *De novo* assembly was performed using SPAdes (version 3.9.0), producing a 20.48-megabase genome comprising 409 contigs and an *N*_50_ of 181.71 kb in 36 contigs.

tBLASTn (https://blast.ncbi.nlm.nih.gov) was used for the identification of *BIO1* orthologs. The amino acid sequence encoded by *ScBIO1* (14, 65) was queried against the translation of whole-genome shotgun (wgs) or nucleotide collection data of single yeast species. In a reciprocal analysis, the yeast-specific best hits with a minimum coverage of 80% were aligned using tBLASTn against the S. cerevisiae CEN.PK113-7D nucleotide sequence to verify the similarity to the *ScBIO1* sequence. The identified putative *BIO1* amino acid sequences (Fig. 3) were pairwise aligned using ClustalΩ (scoring matrix BLOSUM62) (25) to determine amino acid sequence similarities. The *BIO1* structural model was generated by homology modeling using Phyre2 (66).

### Data availability.

The sequencing data and assembly of the Yarrowia lipolytica strain W29 were deposited at NCBI (https://www.ncbi.nlm.nih.gov/) under BioProject accession number PRJNA601425.
